# Nonviral genome engineering of natural killer cells

**DOI:** 10.1186/s13287-021-02406-6

**Published:** 2021-06-16

**Authors:** Gabrielle M. Robbins, Minjing Wang, Emily J. Pomeroy, Branden S. Moriarity

**Affiliations:** 1 Department of Pediatrics, University of Minnesota, Minneapolis, MN 55455 USA; 2 Masonic Cancer Center, University of Minnesota, Minneapolis, MN 55455 USA; 3 Center for Genome Engineering, University of Minnesota, Minneapolis, MN 55455 USA; 4 College of Veterinary Medicine, University of Minnesota, Saint Paul, MN 55455 USA

**Keywords:** Nonviral, NK cells, Genome engineering, Immunotherapy, Cell-based therapy, Transposon, CRISPR/Cas, Nucleofection, Lipofection

## Abstract

Natural killer (NK) cells are cytotoxic lymphocytes of the innate immune system capable of immune surveillance. Given their ability to rapidly and effectively recognize and kill aberrant cells, especially transformed cells, NK cells represent a unique cell type to genetically engineer to improve its potential as a cell-based therapy. NK cells do not express a T cell receptor and thus do not contribute to graft-versus-host disease, nor do they induce T cell-driven cytokine storms, making them highly suited as an off-the-shelf cellular therapy. The clinical efficacy of NK cell-based therapies has been hindered by limited in vivo persistence and the immunosuppressive tumor microenvironment characteristic of many cancers. Enhancing NK cell resistance to tumor inhibitory signaling through genome engineering has the potential to improve NK cell persistence in the tumor microenvironment and restore cytotoxic functions. Alongside silencing NK cell inhibitory receptors, NK cell killing can be redirected by the integration of chimeric antigen receptors (CARs). However, NK cells are associated with technical and biological challenges not observed in T cells, typically resulting in low genome editing efficiencies. Viral vectors have achieved the greatest gene transfer efficiencies but carry concerns of random, insertional mutagenesis given the high viral titers necessary. As such, this review focuses on nonviral methods of gene transfer within the context of improving cancer immunotherapy using engineered NK cells.

## Introduction

Cancer therapy has been revolutionized through the adoptive transfer of tumor-infiltrating lymphocytes (TILs) and genetically engineered T cells. However, clinical efficacy has been largely limited to blood cancers [1]. Natural killer (NK) cells are innate lymphocytes with cytotoxic and inflammatory effector functions unleashed in response to cancer and thus represent an additional cell type of interest for cancer immunotherapy. Moreover, NK cells do not express a T cell receptor (TCR) and thus have not been associated with some of the most detrimental side effects associated with T cell-based therapies, such as graft-vs-host disease (GvHD) and cytokine release syndrome (CRS), making NK cells an attractive candidate for cancer immunotherapy [2]. However, studies to date have shown minimal clinical efficacy with unmanipulated peripheral blood NK cells. Thus, improving NK cell function through genetic modification is of high interest, but primary NK cells display resistance to many gene editing methods that work well in T cells [3–5]. A current consideration for engineered T and NK cell therapy is the method of gene transfer. The vast majority of engineered lymphocytes used in the clinic are made using viral vectors to deliver genetic material [1, 6]. However, high cost of production, long turnaround times, batch to batch variability, and safety concerns have motivated the field to search for nonviral gene delivery and alternative approaches. Here, we review recent advances in the genetic modification of NK cells, with a focus on nonviral strategies.

## NK cell intransigence to genetic modification

Viral vectors including retrovirus and lentivirus have been used extensively to stably deliver genetic material to a broad range of cell types [7]. They have been especially useful in the cell therapy field for the generation of engineered T cell therapies and hematopoietic stem/progenitor cell (HSPC) transplant. In contrast to T cells and HSPCs, NK cells are notoriously difficult to transduce [3, 8]. High expression of pattern recognition receptors (PRRs) activated in response to pathogen- and danger-associated molecular patterns (PAMPs and DAMPs) may in part explain this phenomenon [9, 10]. The RNA genome of retroviruses and lentiviruses is known to activate PRRs including toll-like receptor 3 (TLR3), retinoic acid-inducible gene I (RIG-I), and melanoma differentiation-associated protein 5 (MDA-5) in NK cells [8]. The result is poor NK cell viability and low transduction rates, which hinder the efficacy of this approach for therapeutic use [11].

## NK cell transfection strategies

Transfection is a powerful tool for the deliberate introduction of nucleic acids into cells and can be used to deliver genome editing reagents for gene knockout or delivery of exogenous transgenes. Nonviral methods of transfection most often result in rapid, although transient, transgene expression when compared to viral-based methods [4, 12]. A major concern with the viral modification of NK cells is the risk for insertional mutagenesis given the high viral titers necessary for successful transduction. Conversely, nonviral transfection-based methods do not carry the aforementioned risk, making them more favorable from a safety perspective, especially with an ultimate goal of developing novel immunotherapies. The most common methods of gene transfer using transfection include lipofection and electroporation (Table 1).

**Table 1:** Comparison of nonviral delivery strategies in NK cells

Delivery method	Advantages	Disadvantages	Apparatus
Electroporation Nucleofection	High efficiency Less regulatory constraints cGMP compliant electroporation systems DNA, RNA, or plasmids	NK cells must be expanded and require cytokines Cell viability dependent on cargo (e.g., RNA, DNA) Cargo size affects efficiency	Neon Amaxa BTX MaxCyte
Lipofection	Cost effective Readily available reagents DNA, RNA, or plasmids	Cell viability dependent on cargo (e.g., RNA, DNA) Limited studies Requires optimized conditions of reagents and cell medium	Lipofectamine 2000, 3000

With lipofection, nucleic acids or proteins are encapsulated in cationic liposomes, which fuse with the target cell membrane [13]. Once fused, these liposomes release the cargo directly into the cell. While lipofection of NK cells has historically been used more sparingly, there are new bodies of research utilizing liposome-mediated transfection strategies [13]. One of the earliest studies using lipofection described the transfer of a murine interleukin-2 (IL-2) expressing plasmid into primary NK cells using 1,2-dimyristyloxy-propyl-3-dimethyl-hydroxy ethyl ammonium bromide/dioleoyl phosphatidylethanolamine (DMRIE/DOPE) [14]. IL-2 promotes proliferation and enhances the cytotoxicity of NK cells, including the secretion of granzyme. Investigators found that melanoma xenograft tumors treated with transfected NK cells had significantly higher levels of granzyme A activity [14]. The transformed NK cell line NK-92 (derived from peripheral blood mononuclear cells (PBMCs) of a non-Hodgkin’s lymphoma patient) [15] was lipofected with stem cell factor (SCF) cDNA and found to have significantly greater proliferation and stronger cytotoxicity against a broad range of target tumors when compared to wild-type NK-92 cells [16]. Lipofectamine 2000 has been used to transfect primary NK cells with an activating chimeric antigen receptor (CAR) specific to human epidermal growth factor receptor 2 (HER-2), an oncogene frequently overexpressed in a number of solid tumors. With transfection efficiencies averaging at 60% across ten different donors, these HER-2-specific CAR-NK cells were selectively activated by HER-2-positive tumor cells and eradicated tumor cells in vivo [17]. Youness et al. identified miR-486-5p as a direct regulator of insulin-like growth factor-1 receptor (IGF-1R), which is a known modulator of hepatocellular carcinoma [18]. miR-486-5p was lipofected into primary NK cells, resulting in improved NK cell cytotoxicity through an increase in natural killer group 2D (NKG2D) and perforin expression. Regis et al. found that miR-27a-5p negatively regulates CX_3_C chemokine receptor 1 (CX_3_CR1), which drives NK cells to peripheral tissues, including tumor sites [19]. Investigators utilized Lipofectamine 3000 to transfect primary NK cells with a miR-27a-50 inhibitor and achieved transfection efficiencies of ~30%. Hargreaves et al. compared the transfection efficiency and cellular viability of primary NK cells using different transfection techniques. Both transfection efficiency and cell viability ranged greatly from 0% up to 75% [20]. Most recently, Lipofectamine 2000 and 3000 were compared to a novel transfection reagent method known as charge-altering releasable transporters (CARTs). While lipofectamine-transfected primary NK cells had GFP detection below 1%, CART-transfected NK cells expressed green fluorescent protein (GFP) more efficiently (~10%) and showed improved viability with minimal changes to NK cell phenotype and function [21]. These lipofected cells showed viabilities ranging from 40 to 85%.

Electroporation-based methods are one of the earliest strategies used for nucleic acid delivery in NK cells. Electroporation is a method based on the generation of electrical pulses to induce small, temporary pores in the cell membrane [13]. These pores allow for charged molecules, such as DNA, RNA, and proteins, to move into the cell. Typically, electroporation requires target cells to be dividing in an exponential growth phase in order for nucleic acids to have optimal access to the nucleus. Previous studies demonstrate that primary NK cells require cytokine stimulation and/or expansion using feeder cell lines to allow for sufficient transfection efficiencies and post-electroporation viability [13, 22]. Nucleofection-based methods were developed to allow for efficient gene transfer into the cell nucleus without relying on cell division for nucleic acid transfer into the nucleus. Nucleofection uses the physical methods of electroporation (induction of cell membrane pores) but uses a unique device, known as a Nucleofector, as well as a combination of optimized electrical parameters and cell type-specific reagents [23]. Together, this enables the transfer of molecules directly into the cells’ nucleus, which improves transfection efficiency and faster expression [24]. Using the nucleofector system, transfection efficiencies and cellular viability of NK cells vary greatly. A comparison of multiple transfection methods showed nucleofected NK cells had a transfection efficiency of 60% and post-transfection viability ranging from 50 to 75% [20]. Trompeter et al. aimed to optimize nucleofection of both primary NK cells and the IL-2-dependent NK cell line, NKL. By testing variations in cell number and DNA amount, investigators achieved transfection efficiencies around 50% and cell viability inversely correlated with transfection efficiency due to the toxicity of the DNA [25].

## Transfection of NK cells with in vitro transcribed (IVT) mRNA

While nucleofection methods are still employed, many investigators found that moving away from DNA-based cargo improved viability (Table 2). Carlsten et al. electroporated primary NK cells with mRNA to introduce a high-affinity CD16 and chemokine receptor C-C motif chemokine receptor 7 (CCR7) [26]. Greater than 95% expression was achieved and engineered cells showed substantial migration to chemokine, C-C motif chemokine ligand 19 (CCL19), as well as greater cytotoxicity against antibody-coated lymphoma cells. While electroporation-based methods have been utilized with high efficiencies, viral transduction remains heavily used due to its ability for stable gene transfer. A comparison of mRNA electroporation and lentiviral transduction of the NK-92 cell line showed significantly greater transfection efficiencies and cytotoxicity when compared to mRNA electroporated cells [27]. Interestingly, cord blood NK cells had higher efficiencies when transduced virally, suggesting relevant differences between the NK-92 cell line and primary NK cells [27]. Li et al. found electroporation efficiencies greater than 80% when they introduced an mRNA encoding a CAR receptor against CD19 into rested (unstimulated) and expanded primary NK cells [28]. Both rested and expanded cells transfected with a CD19-CAR showed enhanced cytotoxicity against CD19+ targets when compared to non-transfected cells. Similarly, NK-92 cells have also been transfected with mRNA to express a CD19-CAR, chemokine receptor CCR7, as well as other reporter genes such as enhanced GFP (eGFP), yellow fluorescent protein (YFP), and Azuride [12]. Both viability and transfection efficiencies achieved were between 50 and 60%, with CD19-CAR-transfected cells showing improved cytotoxicity against CD19+ cell lines during in vitro killing assays. Importantly, delivery of activating receptors with mRNA or plasmid DNA results in transient expression, so alternative approaches are necessary to achieve stable expression of transgenes.

**Table 2:** Comparison of NK cell engineering reagents

Cargo	Efficiency	Viability	Advantages	Disadvantages	Therapeutic uses
Transient mRNA DNA	Up to 99%	Poor to good	Rapid expression High efficiency	Transient—no stable genomic integration Cell viability dependent on cargo (e.g., RNA, DNA)	Transient CAR mRNA Knockout of genes that suppress or inhibit NK cell function Knock-in of activating receptors or genes that promote NK cell function
Transposon	Up to 80%	Poor to good	Cost effective Large cargo capacity Stable integration	Potential insertional mutagenesis Transposon must be delivered as DNA	Large cargo delivery (e.g., CAR in combination with activating receptors or cytokines)
Cas9 Base editor Prime editor	Up to 100%	Poor to excellent	High precision High efficiency Large-scale insertion or deletion	Potential off target editing Indels and translocations	Knockout genes that suppress or inhibit NK cell function Knock-in of activating receptors or genes that promote NK cell function Treating patients bearing disease caused by a single base pair mutation

## NK cell engineering with DNA transposons

A common strategy for stable, nonviral gene delivery is the use of DNA transposons (Table 2). Transposons, also known as transposable elements, are naturally existing repetitive DNA sequences that are capable of mobilizing from one location to another in the genome [29]. When used for gene delivery, DNA transposons are generally a two-component system, with a transposon vector containing sequences to be mobilized flanked by terminal inverted repeats (TIRs) and a transposase enzyme that identifies the TIRs and excises and re-integrates the transposon [30]. Since their discovery 70 years ago by Barbara McClintock, the “cut and paste” mechanism of transposons has been used as a genetic tool for multiple purposes, ranging from genetic screening to insertional mutagenesis and transgenesis [29, 31]. As we enter the era of gene therapy and personalized medicine, transposons have been used extensively as an alternative to the viral vector system for engineering human cells. Recently, transposon-engineered induced pluripotent stem cells (iPSCs) and T cells have been used in clinical trials, making the transposon system one of the most promising nonviral vector systems for stable gene transfer [30, 32].

There are three major superfamilies of transposons commonly used for gene transfer in human cells, namely Tc1/mariner, piggyBac (PB), and hAT [29]. The most extensively studied transposon system for gene transfer, the Sleeping Beauty (SB) transposon system, belongs to the Tc1/mariner superfamily [29]. Since its discovery and molecular reconstruction from the genomes of salmonid fish [33], SB has undergone improvements through the generation of hyperactive mutants and transposon donor vector optimization [34, 35]. The most hyperactive variant developed so far, SB100X, is shown to have a 100-fold improvement of integration efficiency compared to the original SB transposase and is comparable to that of a viral vector system [35]. For example, in human CD34+ hematopoietic cells, SB100X is able to achieve up to 50% integration efficiency, compared to 40% with lentiviral vectors [35, 36]. SB integration has a consensus target site TA. High-resolution genome-wide mapping showed SB integration favors introns, transcriptional units, upstream regulatory sequences, and microsatellite repeats [37, 38]. PB is another well-developed transposon system for stable gene transfer that was isolated from the cabbage looper moth [29, 39]. The PB system shares a similar transposition mechanism with SB, and its efficiency has also been significantly improved by hyperactive mutant screening and transposon/transposase vector optimization [39, 40]. The current hyperactive PB (super-PB) also exhibits a comparable efficiency of transposition to viral vectors [39]. PB also exhibits a non-random integration pattern, but with slightly different preferences compared to SB. PB has a TTAA consensus target site and shows a tendency to integrate into introns, transcriptional start sites, and long terminal repeat elements [37, 38]. As a novel representative of the hAT family, the TcBuster transposon originated from the red flour beetle and is a rising star for gene transfer [41]. TcBuster was shown to be highly active in human cell lines, including HEK-293 and HeLa cells, and has a comparable transposition efficiency to PB and SB [42, 43]. TcBuster has an integration pattern that is slightly different from SB and PB. TcBuster favors a TA integration site, and as for genomic elements, it slightly favors transcription units, CpG islands, and transcription start sites [37, 38].

Engineering NK cells using transposable elements has gained fairly limited attention and even fewer publications thus far. Among these published studies, NK-92 is almost exclusively used [27, 44]. For instance, in collaboration with the Kaufman laboratory, we previously utilized NK-92 cells to screen mesothelin-specific CAR constructs to enhance NK cell activity [45]. We successfully expressed a panel of novel CAR architectures in NK-92 cells using PB or SB systems and demonstrated improved anti-tumor activities of CAR-expressing NK-92 cells when co-cultured with mesothelin-expressing targets [45]. Another study focused on expressing a CD73-specific CAR in NK-92 cells [46]. Using the PB system, Matosevic’s group delivered a CD73-CAR construct to NK-92 cells and showed potent killing ability against both solid tumor target cells and humanized CD73+ lung cancer patient-derived xenograft (PDX) models [46].

As an alternative to transfecting NK cells directly with transposon systems, transfecting iPSCs and differentiating them into NK cells allows for the circumvention of low transfection efficiency of plasmids in primary NK cells [45, 47]. Using this approach, Li et al. were able to express optimized mesothelin-CAR constructs using a super-PB system in human iPSCs, followed by differentiation into NK cells [45]. Like NK-92, stable expression of mesothelin-CAR was achieved in iPSCs, and subsequent functional assays indicated enhanced tumor specificity and killing in iPSC-derived CAR-NK cells [45].

**Fig. 1:**
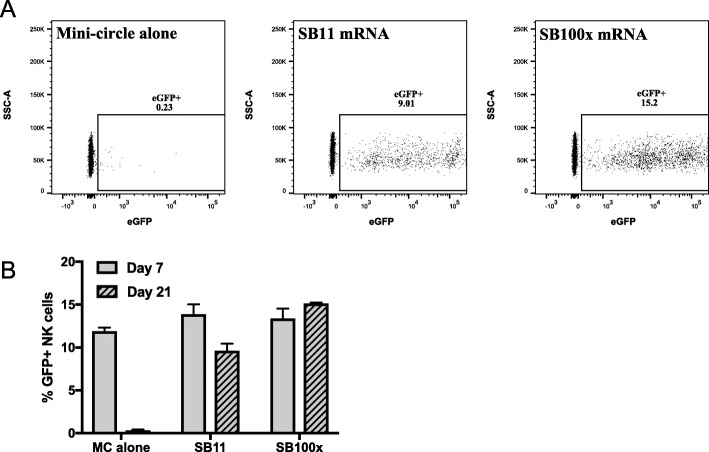
SB mRNA and minicircle delivery of GFP to primary human NK cells. Primary human NK cells (*n* = 2 independent donors) were isolated from peripheral blood and expanded using membrane-bound interleukin-21 (mbIL21)-expressing K562 feeder cells. After expansion, NK cells were electroporated with minicircle (MC) plasmid expressing eGFP alone or in combination with SB11- or SB100X-encoding mRNA. **A** Representative flow plots of eGFP expression 21 days after electroporation. **B** eGFP expression from 2 NK cell donors 7 and 21 days after electroporation

Our finite understanding of NK cells in general and their notorious aversion to transgene uptake and expression are likely to blame for the limited study of transposable elements in primary NK cells. Transposons, especially SB and PB, have been used extensively in delivering CARs to T cells for treating both hematological and solid tumors, with targets including CD19 [48], CD33 [49], CD133 [50], epidermal growth factor receptor (EGFR) [51], mesothelin [52], and glypican 3 (GPC3) [53]. Among them, a CD19-CAR and a signaling lymphocyte activation molecule family member 7 (SLAMF7)-CAR, both generated using the SB system, have already entered phase I/II clinical trial for treating leukemia or lymphoma [54, 55] and multiple myeloma (MM) [56], respectively. These achievements in T cells offer both insight and a basis for applying the concept in primary NK cells. With a better understanding of NK isolation and expansion, as well as the improvements in SB technology through hyperactive mutant generation (SB11, SB100X) and transposon donor vector optimization (pT4) [34, 35], it is feasible to expand and apply transposon systems for engineering primary NK cells. To this end, our group performed proof-of-principle experiments delivering mRNA encoding SB11 or SB100X in combination with a minicircle DNA transposon encoding GFP to feeder cell-expanded primary human NK cells (Fig. 1). We show stable expression of GFP 21 days after electroporation, with 15% efficiency using SB100X, suggesting this is a viable approach for nonviral, transposon-based gene delivery to NK cells.

Because of their higher gene transfer efficiencies, viral vector systems, including lentivirus, adeno-associated virus (AAV), and retrovirus, are still the preferred vector system used in NK cell engineering. Nevertheless, transposon systems are shown to overcome many limitations of the viral vector systems. Larger cargo size is one of the most prominent features transposon systems have over viral vectors [57]. Transposons have at least 9 kb of cargo capacity, while the largest carrying capacity for viral vectors has an 8-kb size limitation [57]. Moreover, SB was shown to have significantly lower integration rates than that of almost all the common viral vectors, with avian sarcoma leukosis virus (ASLV) being the only exception and sharing a similar rate as SB [37]. And though PB demonstrated a slightly higher integration frequency than SB, the rate is still lower than that of HIV-1 [38]. Together, this indicates a greater safety profile of transposon systems than that of integrating viral vector systems. Additionally, transposon systems are more cost-effective and easier to produce and purify [29]. With the recent improvements of gene transfer efficiencies in transposon systems, especially that of SB and PB, the efficiencies are comparable to that of viral vectors [35, 39], making transposon systems a very promising gene editing tool for NK engineering.

## NK cell engineering with Cas9, base editors, and prime editors

The expression of an array of inhibitory receptors and checkpoint molecules that can be upregulated in the tumor microenvironment (TME) has made clustered regularly interspaced short palindromic repeats (CRISPR)/CRISPR-associated protein 9 (Cas9)-based gene editing in NK cells a goal for scientists trying to augment NK cell anti-tumor efficacy [5] (Table 2). Early efforts to use CRISPR/Cas9 in primary NK cells used Cas9 expressed via a DNA plasmid or mRNA. This often resulted in low transfection efficiencies [58]. A method has recently been described to efficiently and reliably target genes for knockout with CRISPR/Cas9 in primary human NK cells using chemically modified guide RNAs and Cas9 mRNA [22]. Using this method, Pomeroy et al. targeted NK inhibitory genes (ADAM metallopeptidase domain 17) *ADAM17* and (programmed cell death 1) *PDCD1* for knockout and developed a method for targeted integration using recombinant adeno-associated virus (rAAV) as a donor template for homology-directed repair (HDR). Gene knockout and knock-in efficiencies in this study reached 90% and 75%, respectively, equivalent to reports of analogous approaches in primary T cells. Other groups have solved the problem of low transfection efficiency by delivering Cas9 and guide RNAs as ribonucleoprotein (RNP) complexes, as this approach has shown high editing rates in primary T cells [59]. Through the use of RNPs, Rautela et al. were able to achieve editing efficiencies of up to 75% across a number of genes in primary NK cells [60]. Others have used RNPs to efficiently knockout transforming growth factor beta receptor 2 (*TGFBR2*) in primary NK cells, as transforming growth factor beta (TGFβ) is a major NK cell inhibitor [59]. Electroporation of cells with RNP complex to knockout *TGFBR2* resulted in a 60% reduction in mRNA expression. Recently, Nguyen et al. developed methods to improve the efficiency of site-specific CRISPR/Cas9-based gene delivery, using Cas9-RNPs and DNA templates for HDR containing truncated Cas9 target sequences (tCTSs). The tCTSs associate with the Cas9-RNPs and are thus shuttled to the nucleus, which enhances HDR efficiency [61]. Using this approach, they achieved over 15% transgene delivery to NK cells.

Base editors (BEs) are another gene editing tool that takes advantage of the CRISPR/Cas system. BEs are composed of a catalytically inactive Cas9 protein fused to a DNA deaminase domain [62]. Unlike Cas9 nuclease, this feature enables precise introduction of targeted single nucleotide changes without introductions of double-strand breaks (DSBs) or the need for a DNA donor molecule [63]. There are two types of base editors to date, adenine base editor (ABE) and cytosine base editor (CBE), and collectively, they can achieve all possible transition mutations (A->G for ABE and C->T for CBE) [64, 65]. Due to its relatively recent development, applications of BE in the immunotherapy context are very limited. Studies using BE in T cells have just started emerging; however, applications in NK cells are still lacking. Last year, Webber and Lonetree et al. reported a multiplex knockout of T cell receptor alpha constant (*TRAC*), ß-2 microglobulin (*B2M*), and *PDCD1* in CD19 CAR-T cells using both CBE base editors [66]. In this study, they achieved higher than 90% editing efficiency across all 3 target genes, at both DNA and protein levels [66]. Additionally, Zhang’s group reported a down-regulation of PD-1 expression in CAR-T cells using ABE [67]. By altering the coding sequencing of N74 in the *PDCD1* gene, they reduced asparagine (N)-linked glycosylation of PD-1 protein. This modification reduced its inhibitory effect on CAR-T cells. These studies serve as a proof of principle for using both ABE and CBE in T cells, and potentially NK cells, given the shared characteristics and functioning mechanisms between these two cell types.

Last year, Liu’s team reported prime editor (PE), further expanding our ability to precisely engineer DNA without inducing DSBs or a need for DNA donor molecules [62, 68]. PE uses a reverse transcriptase fused to dead Cas9 (dCas9) and prime editing guide RNA (pegRNA) containing a sequence to be introduced. PE is capable of introducing all possible transversion and transition mutations, as well as small insertions and deletions [68]. At its current stage, PE has only been tested in a very limited number of mammalian cells, including 293T and K562, with up to 70% and 30% editing efficiency, respectively [68]. However, sharing a similar mechanism as BE, it is worth trying this technology in NK cells. If successful, this would greatly enhance our toolbox for editing NK cells using nonviral approaches.

## Summary

NK cells have shown great promise as a cell-based therapy for cancer, but there is much work to be done to improve their anti-tumor efficacy. Recent advances in NK cell engineering, especially using nonviral methods, may unleash the therapeutic potential of NK cells as a cancer therapeutic. NK cell effector functions are regulated by an array of activating and inhibitory receptors. Cancer cells can evade NK cell detection by interacting with these receptors directly or by secreting immunosuppressive molecules. Therefore, genetic manipulation of NK activating or inhibitory receptors may augment anti-tumor activity. The most common nonviral delivery strategies for gene editing NK cells are lipofection and electroporation. While lipofection can be more cost-effective than electroporation, it is clear that electroporation is the more efficient strategy and is well suited for clinical translation. Of particular interest are genome engineering reagents that confer stable gene delivery like transposons and gene editing tools that do not induce DSBs, such as BE and PE. As we continue to learn more about why NK cells resist transgene delivery, we can find new and creative ways to work around them and increase the efficiency of these techniques.

## Materials and methods

### NK cell isolation and expansion

PBMCs from de-identified healthy human donors were obtained by automated leukapheresis (Memorial Blood Centers, Minneapolis, MN, USA). CD56+CD− NK cells were isolated by negative selection using the EasySep Human NK Cell Enrichment Kit (STEMCELL Technologies). After isolation, NK cells were expanded by co-culture with irradiated (100 Gy) mbIL21- and 4-1BB ligand (41BBL)-expressing K562 feeder cells as described previously [21].

### Electroporation of expanded NK cells

Expanded NK cells were pelleted and resuspended at 3 × 10^7^ cells/mL in T buffer (Neon Transfection System Kit; Thermo Fisher Scientific). One microgram of MC plasmid DNA and 1 μg SB11 or SB100X mRNA were added to 10 μL (3 × 10^5^ cells) on ice. This mixture was electroporated with the Neon Transfection System (Thermo Fisher Scientific) using two pulses of 1850 V and 10-ms pulse width. NK cells were re-expanded with feeder cells for 21 days.

### Flow cytometry

The following antibodies were used: allophycocyanin (APC)-conjugated anti-CD56 (clone REA196; Miltenyi Biotec), phycoerythrin (PE)-conjugated anti-CD3 (clone SK7; BD Biosciences), and SYTOX Blue dead cell stain (Thermo Fisher). Flow cytometry assays were performed on LSR Fortessa flow cytometers (BD Biosciences), and data were analyzed using FlowJo version 10.4 software (FlowJo).

## Data Availability

Not applicable

## Abbreviations

41BBL: 4-1BB ligand
5′UTR: 5′ untranslated region
AAV: Adeno-associated virus
ABE: Adenine base editor
ADAM17: ADAM metallopeptidase domain 17
APC: Allophycocyanin
ASLV: Avian sarcoma leukosis virus
B2M: ß-2 microglobulin
BEs: Base editors
CAR: Chimeric antigen receptor
CARTs: Charge-altering releasable transporters
Cas9: CRISPR-associated protein 9
CBE: Cytosine base editor
CCL19: C-C motif chemokine ligand 19
CCR7: C-C motif chemokine receptor 7
cDNA: Complementary DNA
CRISPR: Clustered regularly interspaced short palindromic repeats
CRS: Cytokine release syndrome
CX_3_CR1: CX_3_C chemokine receptor 1
DAMPs: Danger-associated molecular patterns
dCas9: Dead Cas9
DMRIE: 1,2-Dimyristyloxy-propyl-3-dimethyl-hydroxy ethyl ammonium bromide
DOPE: Dioleoyl phosphatidylethanolamine
DSBs: Double-strand breaks
eGFP: Enhanced green fluorescent protein
EGFR: Epidermal growth factor receptor
GFP: Green fluorescent protein
GPC3: Glypican 3
GvHD: Graft-vs-host disease
hAT: hobo/Ac/Tam3
HDR: Homology-directed repair
HER-2: Human epidermal growth factor receptor 2
HSPC: Hematopoietic stem/progenitor cell
IGF-1R: Insulin-like growth factor-1 receptor
IL-2: Interleukin-2
iPSCs: Induced pluripotent stem cells
IVT: In vitro transcribed
mbIL21: Membrane-bound interleukin-21
MC: Minicircle
MDA-5: Melanoma differentiation-associated protein 5
MM: Multiple myeloma
NK cells: Natural killer cells
NKG2D: Natural killer group 2D
PAMPs: Pathogen-associated molecular patterns
PB: PiggyBac
PBMCs: Peripheral blood mononuclear cells
PDCD1: Programmed cell death 1
PDX: Patient-derived xenograft
PE: Prime editor
PE: Phycoerythrin
pegRNA: Prime editing guide RNA
PRRs: Pattern recognition receptors
RIG-I: Retinoic acid-inducible gene I
RNP: Ribonucleoprotein
SB: Sleeping Beauty
SCF: Stem cell factor
SLAMF7: Signaling lymphocyte activation molecule family member 7
tCTSs: Truncated Cas9 target sequences
TGFBR2: Transforming growth factor beta receptor 2
TGFβ: Transforming growth factor beta
TILs: Tumor-infiltrating lymphocytes
TIRs: Terminal inverted repeats
TLR3: Toll-like receptor 3
TME: Tumor microenvironment
TRAC: T cell receptor alpha constant
YFP: Yellow fluorescent protein
